# Insights into Acute Pancreatitis Associated COVID-19: Literature Review

**DOI:** 10.3390/jcm10245902

**Published:** 2021-12-16

**Authors:** Yasameen E. Muzahim, David C. Parish, Hemant Goyal

**Affiliations:** 1 Department of Internal Medicine, Atrium Health-Navicent, Macon, GA 31201, USA; 2 Department of Internal Medicine, Mercer University School of Medicine, Macon, GA 31207, USA; PARISH_DC@mercer.edu; 3 The Wright Center for Graduate Medical Education, Scranton, PA 18501, USA; doc.hemant@yahoo.com

**Keywords:** coronavirus disease 2019, SARS-CoV-2, acute pancreatitis

## Abstract

Severe acute respiratory syndrome coronavirus-2 (SARS-CoV-2) primarily affects the lungs, causing respiratory symptoms. However, the infection clearly affects all organ systems including the gastrointestinal system. Acute pancreatitis associated with coronavirus disease 2019 (COVID-19) has been widely reported Recent studies have discussed pancreatic compromise incidentally in asymptomatic patients, or in a form of clinical symptoms such as abdominal pain, nausea, or vomiting, which is further reflected in some cases with abnormal serum lipase and amylase levels It was suggested that upregulation of angiotensin-converting enzyme II cell receptors or inflammatory cytokines play a major role in predisposing pancreatic injury in SARS-CoV-2 positive patients To date, there is insufficient data to establish the causality of acute pancreatitis in SARS-CoV-2 infected cases. In this paper, we organize recent studies conducted to observe the frequency of acute pancreatitis associated with COVID-19 cases while highlighting present hypotheses, predisposing factors, and their effect on the outcome, and point to gaps in our knowledge.

## 1. Introduction

In December 2019, coronavirus disease 2019 (COVID-19) caused by severe acute respiratory syndrome coronavirus 2 (SARS-CoV-2) was first detected in Wuhan City, China. Since then, COVID-19 has become a world-wide pandemic affecting the lives of millions of people and raising mortality rates due to viral infection or its sequelae [1]. As reported by the World Health Organization in June 2021, there have been over 170 million confirmed cases of COVID-19 globally, including over 4 million deaths [2]. It was initially found that it causes a broad range of upper and lower respiratory symptoms such as cough and dyspnea in addition to systemic manifestations of fever, loss of taste and/or smell, myalgia, and arthralgia. Extrapulmonary compromise involving gastrointestinal (GI), pancreatic, and hepatobiliary among other systems has been reported. Most common GI symptoms presented were abdominal pain, nausea with/without vomiting, diarrhea, and anorexia [3].

Pancreatic injury can manifest from an asymptomatic elevation of amylase and lipase to severe acute pancreatitis (AP) [4]. In this paper, we discuss the clinical characteristics of AP-associated COVID-19 infections to uncover the related risk factors and to investigate AP effect on prognosis in COVID-19 infected patients. 

## 2. Methods

A comprehensive literature review was conducted through Ovid and PubMed advanced search engine. All relevant English-written articles were used in the medical subject heading (MeSH) terms.

Ovid: “COVID-19.mp.” OR “exp COVID-19/:” OR “SARS-CoV-2.mp.” OR “exp SARS-CoV-2/” OR “exp SARS Virus/” OR “Lipase.mp.” OR “exp Lipase/” OR “amylase.mp.” OR “exp Amylases/” OR “exp Pancreas/” OR “exp Pancreatitis/” OR “pancreatic enzymes.mp”.

PubMed: “COVID-19” OR “coronavirus 2019” OR “SARS-CoV-2” AND “pancreatitis” OR “elevated lipase” OR “elevated amylase.” 

**Figure 1:**
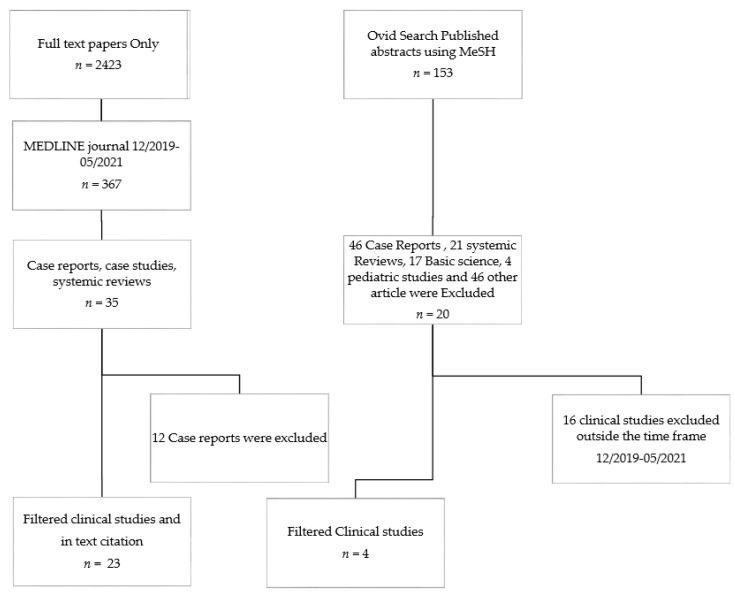
Flow chart of the selected literature work included in the systemic review.

As illustrated in Figure 1, 174 published papers combined from our search engines were identified. Case reports were excluded as they carry a high risk of bias, and we chose to exclude the filtered case studies and systemic reviews because we had a sufficient data set for our review. Our literature references, as indicated in Table 1, were selected from the filtered results published between December 2019 and May 2021 in addition to their in-text citation literature sources. Additionally, several articles published between 2000–2016 were cited to highlight other aspects on the etiology, diagnosis, or pathogenesis of AP. 

## 3. Results

A retrospective cohort study was conducted on 52 patients with COVID-19 pneumonia admitted to Zhongnan Hospital of Wuhan University from 20 January to 28 February 2020. The most common initial symptoms were fever, fatigue, dry cough, myalgia, and dyspnea. Less common symptoms were headaches, dizziness, abdominal pain, diarrhea, nausea, and vomiting. Out of 52 patients, there were nine patients (17%) who presented with pancreatic injury, as defined by elevated amylase or lipase. Patients with pancreatic insult had an average age of 55 years. Other laboratory test results in this group showed a decrease in lymphocytes and an increase in hepatic and inflammatory indicators [5]. 

Another retrospective observational cohort study was conducted on patients 18 years or older admitted to 12 hospitals within the Northwell Health System from 1 March to 1 June 2020, during the COVID-19 pandemic in New York. During this period, 48,012 patients were hospitalized, and 11,883 of 48,012 (24.75%) were COVID-19 positive on admission [6]. The COVID-19 pneumonia cases were not described in this study. Patients were identified as presenting with acute pancreatitis on admission if they met all three of the following criteria: lipase level greater than 3 times the upper limit of normal, computed tomography or magnetic resonance imaging showing pancreatitis, and characteristic upper abdominal pain at admission [7]. A total of 189 of 48,012 (0.39%) met criteria for a diagnosis of pancreatitis, and 32 of 189 (17%) were COVID-19 positive, yielding a point prevalence of (0.27%) of pancreatitis among the 11,883 patients with COVID-19 associated hospitalizations. With respect to outcomes, patients with pancreatitis who were also COVID-19 positive were more likely to require mechanical ventilation and had longer lengths of hospital stay compared to patients with pancreatitis without COVID-19 [6]. In a systematic search paper [8] citing the above retrospective study, the suggestion was made to conduct autopsies on a series of patients with COVID-19 with and without concomitant clinically evident acute pancreatitis, as it might help in revealing the mechanisms behind this possible, yet unproven, association.

A retrospective cohort study was conducted to assess the impact of COVID-19 on pancreatic injury in a US population. A total of 71 hospitalized patients across six US centers (two tertiary and four community hospitals) with COVID-19 who had lipase levels measured were included. Hyperlipasemia was defined as an elevated lipase level above the upper limit of normal (>60 U/L). Poor outcomes included intubation, intensive care unit (ICU) admission, and death. Demographic data, presenting symptoms, imaging, and laboratory data were obtained from medical records. The study did not mention if study subjects had pulmonary or other organ involvement. The mean age of all patients was 64.9 years. Thirty-nine patients (53.5%) were women, and the average body mass index of all patients was 29.5 (SD ± 6.6). Nine patients (12.1%) had hyperlipasemia >60 U/L on admission. Gastrointestinal symptoms were common among the nine patients with hyperlipasemia, including five (55.6%) with nausea, six (66.7%) with anorexia, three (33.3%) with general abdominal discomfort, and five (55.6%) with diarrhea. None of the cases showed any features characteristic of AP on abdominal CT [9].

A retrospective analysis was conducted on patients diagnosed with COVID-19 from 1 January 2020 to 15 February 2020 in Wuhan Tongji Hospital and Wuhan Jin Yin-tan Hospital. Patients’ hospital admission data, laboratory tests, and imaging tests from clinical electronic medical records were reviewed. Severe COVID-19 was defined when patients had one of the criteria such as shortness of breath and respiratory frequency ≥30/min, finger pulse oximeter oxygen saturation at rest of 93% or less, or oxygenation index of 300 mm Hg or less. In this study cohort, 121 COVID-19 patients were included (46 women, 75 men). In 54 mild cases, one (1.85%) had increased levels of both amylase and lipase. In patients with severe COVID-19, 12 of 64 (17.91%) and 11 of 64 (16.41%) had increased amylase or lipase levels, respectively. In addition, some critically ill patients had already developed pancreatic injury before admission, and drug-induced pancreatitis should be considered a possibility because of the history of taking nonsteroidal anti-inflammatory drugs, glucocorticoids, and other experimental therapies in some of the cases [10].

In a multivariable-adjusted model, a cohort study was conducted at Rush University Medical Center in Chicago, Illinois, between 12 March and 3 April 2020. Of the 294 COVID-19-positive patients admitted to the hospital, 83 patients (18%) were tested for lipase, and 14 of the 83 (16.8%) had elevated lipase levels. A significant predominance of men was observed. There were 26 male patients of 69 total patients (38.8%) in the low lipase group compared with the 11 of 14 patients (78.6%) with elevated lipase group (*p* = 0.009). However, no other significant difference was observed in the demographics of the two groups except higher symptoms of nausea or vomiting reported in 52 of 69 patients (75.4%) in the low lipase group versus 6 of 14 patients (42.9%) in the high lipase group (*p* < 0.025). There was no other significant difference in recorded gastrointestinal symptoms such as abdominal pain or diarrhea between the two groups. Moreover, the elevated lipase level in both groups was significantly associated with higher rates of admission to the ICU and intubation after adjusting other confounders such as age, sex, BMI, history of diabetes, and history of hypertension [11]. 

Mass General Brigham Healthcare Institutional Review Board approved a retrospective study [12] on all the adult patients older than 18 years of age who were admitted with a diagnosis of COVID-19 and AP from 1 February to 30 June 2020. Results are presented using pooled data from Massachusetts General Hospital, Brigham and Women’s Hospital Brigham and Women’s Faulkner Hospital, North Shore Medical Center, and Newton–Wellesley Hospital. COVID-19 disease was confirmed by detecting SARS-CoV-2 nucleic acid in throat swab by reverse-transcription PCR assay. Lipase level was tested in 985 COVID-19 patients. A further search was conducted to include patients with concurrent diagnoses of AP and COVID-19. A total of 17 patients were found eligible for the study. Out of 17, nine patients (52.9%) were primarily hospitalized for coronavirus disease-associated acute respiratory distress syndrome requiring intubation and mechanical ventilation. These patients developed AP after a median 22.5 days (range 13–76 days) from the onset of COVID-19 symptoms. The remaining eight patients (47%) presented with symptomatic AP on admission. Of eight, three (37.5%) patients developed respiratory and constitutional symptoms of COVID-19 illness, one (12.5%) before the diagnosis of AP, and two patients (25%) developed fever and cough after 3 days of hospitalization. The median peak lipase among mechanically ventilated patients was higher (661 vs. 236 U/L). One patient in each group did have elevated lipase, but their clinical course and CT imaging were characteristic of AP. In the first cohort of nine patients, five (55.6%) underwent CT imaging of the abdomen. Typical findings of AP were appreciated in three (33.3%) patients while the pancreas was reported normal in other two patients (22.2%). Necrotizing pancreatitis was present in one patient (11.1%). Among the remaining four of nine patients (44.4%), CT was not performed due to hemodynamic instability in three patients (33.3%), and the fourth patient (11.1%) developed AP with typical symptoms. In the second cohort of eight patients, seven (87.5%) underwent CT. Five patients (62.5%) had typical radiological finding of AP; however, the pancreas was read as normal in the remaining two patients (25%). 

Moreover, a retrospective study was conducted involving 42 COVID-19 patients who were diagnosed using real-time PCR and were admitted to a tertiary care hospital in New Delhi. Serum amylase and serum lipase levels were measured. Serum amylase was elevated in 14 of the 42 patients (33%). Serum lipase was elevated in 7 out of 29 patients (24.1%). Mortality was seen in 18 patients (42.8%). Serum amylase or lipase did not correlate with severity of COVID-19 or its mortality. Although the prevalence of hyperamylasemia and elevated amylase in patients of COVID-19 were 33% and 24.1%, pancreatic injury is not statistically significant in relation to the severity or outcome of COVID-19 [13].

A prospective observational single center study was conducted in the respiratory unit at San Paolo Hospital in Milan, Italy from 1 April to 30 April 2020 [14]. One hundred and ten consecutive patients met the inclusion criteria of the study to be at an age equal to or greater than 18 years, both genders included, recent coronavirus infection confirmed by real-time polymerase chain reaction, and clinically diagnosed with COVID-19 per WHO guidelines. This, in addition to chest CT scans, confirmed lung compromise. None of the patients complained of abdominal pain. Of the 110 patients, 14 (12.7%) had diarrhea and 3 (2.7%) had nausea/vomiting. There was only one patient who experienced all three symptoms. None of the patients studied developed clinical signs consistent with acute pancreatitis. The serum levels of amylase, lipase, total bilirubin, direct bilirubin, alanine aminotransferase, aspartate aminotransferase, γ-glutamyl transpeptidase, and C-reactive protein were recorded during the patients’ initial observation. Results showed 27 of 110 patients (24.5%) had amylase values above 53 IU/L and 18 patients of 110 (16.4%) had lipase values above 300 IU/L. Only one patient of the included patients (0.9%) had values of both amylase and lipase more than 3x the upper normal limit. There was also no statistically significant difference in amylase and lipase serum activities in patients who complained of GI symptoms and in those who did not. 

From a different perspective, studies have been conducted to assess contributing risk factors for AP associated COVID-19 and disease outcome. In a cohort study of 35 patients evaluated at the Royal Liverpool University Hospital between 14 March and 30 April 2020, registered with the Liverpool University Hospitals NHS, cases were identified by searching admission diagnoses or radiology requests and reports for AP. In data extracted from patient and radiology records of contrast-enhanced computed tomography (CECT) images, as reported by an expert pancreatic radiologist, 25 of 35 patients who presented with acute pancreatitis were negative for SARS-CoV-2 and were excluded. Of the 10 patients who tested positive for SARS-CoV-2, a further five were excluded because they presented with a clearly defined etiology of AP (such as choledocholithiasis) with confirmed AP. The five remaining patients were young adult males with a median age of 42 years, overweight or obese with evidence of metabolic distress. Serum amylase was elevated with abdominal CT to confirm the diagnosis, but importantly they had no prior pancreas symptoms. This study postulated that the combination of male sex, abdominal pain, metabolic stress, and CT findings of remarkable for pancreatic inflammation reflects AP in patients infected with SARS-CoV2. Additionally, patients with pre-existing metabolic syndrome can be predisposed to AP considering the high body mass indices as in this study [15]. The small number of subjects render these conclusions interesting and worthy of further study.

Additionally, the clinical outcomes in patients with COVID-19 were observed from seven hospitals in Minnesota during a 4-month period. A retrospective analysis of patients managed within M Health Fairview in Minnesota between 1 March and 30 June 2020 was conducted to study the occurrence of AP. Inpatient adults with an established diagnosis of AP, who underwent polymerase chain reaction testing for SARS-CoV-2 on nasopharyngeal swabs during the index admission or within 14 days before hospitalization, were included. A total of 75 out of 339 patients with AP (22%) had documented PCR testing for SARS-CoV-2 of whom 14 patients (18.7%) tested positive for COVID-19. No significant differences were observed in relation to age, gender, or body mass index between the COVID-19–positive and COVID-19 negative cohorts. Mortality was significantly higher in 3 of 14 patients with AP and coexisting COVID-19 compared to the 1 of total 61 patients with AP and negative SARS-CoV-2 PCR (*p* = 0.004) [16]. This study has limitations given the poorly detailed collected data in COVID-19 positive patients, such as inflammatory markers, which might contribute to the severity of the disease stratification. The study was also based on observations from different hospitals with no standardized data documentation. 

Another cross-sectional study was of a prospective observational cohort covering all COVID-19 patients admitted to two Dutch university hospitals between 4 March and 26 May 2020 [17]. The study protocol was approved by the IRB of Amsterdam Medical Center. All patients with confirmed COVID-19, with or without AP, were included. An experienced abdominal radiologist reviewed the CT scans of patients with elevated amylase/lipase levels during hospital admission. Confirmed COVID-19 was defined as a positive result on high-throughput sequencing or RT-PCR assay of nasal and pharyngeal swab specimens and/or characteristic findings on chest CT. A total of 433 patients with COVID-19 were included. ICU admission was required in 160 patients (37%), with a median ICU stay of 11 days (range 6–18 days). Serum amylase/lipase levels were measured in 160 of 433 patients (37%). Upper abdomen CT was performed on 368 of 433 patients (85%), chest CT including the upper abdomen was performed on 316 patients of 368 (73%), and abdominal contrast-enhanced CT was performed on the remaining 52 patients (12%). There were no abnormalities on CT suggesting AP in patients with normal pancreatic enzymes. Of 368 patients, 15 patients (4%) had elevated amylase/lipase levels of 3x ULN during hospital admission, of which 11 patients (73%) had CT performed. However, seven patients did not report abdominal pain, and the CT did not show characteristic findings of AP. Therefore, only eight of 368 patients (2%) met the criteria of AP [7]. Only five patients were diagnosed with AP based on two criteria: abdominal pain and serum amylase/lipase levels of 3x ULN. One of eight patients met all criteria. In two patients, the diagnosis was based on elevated serum amylase/lipase levels 3x ULN with CT finding of AP. It was not feasible to evaluate abdominal pain due to sedation/intubation. Three patients of the eight who were initially suspected for COVID-19 related pancreatitis had other etiology of acute pancreatitis (two biliary and one post-ERCP), leaving five patients of 433 (1.2%) with potential COVID-19 related acute pancreatitis.

## 4. Discussion

COVID-19, caused by SARS-CoV-2, is a viral disease that affects the entire body. The primary organ system affected is the respiratory tract, including the upper and lower respiratory tract. There is increasing evidence of its effect on extrapulmonary systems, including GI. There have been a number of studies demonstrating pancreatic involvement, including acute pancreatitis presentation, in patients with COVID-19. Given the increasing number of cases reported with GI symptoms in COVID-19 positive patients, studies have focused on observing the extent of GI compromise especially when it comes to pancreatic injury. 

The studies reviewed in this paper use a range of subject selection criteria and definitions of pancreatic injury as illustrated in Table 1; there is no benefit to combining these diverse study groups, however, we attempted to present the results in two main subgroups. The first subgroup discussed studies focused on the association of COVID-19 and the elevated levels of lipase and/or amylase. The second subgroup presented COVID-19 admissions in patients with established diagnosis of acute pancreatitis. Consistent across the studies is that pancreatic involvement is present and in low frequency in those studies with a non-COVID-19 group for comparison. Further studies are needed to clarify the rates of pancreatic involvement and the contribution of this to the overall burden of illness in COVID-19 patients.

Although the pathogenesis is still being investigated, hypotheses have suggested that SARS-CoV-2 enters the cell by binding to the angiotensin-converting enzyme II (ACE-II) receptor and begins viral replication. Although ACE-II is expressed mostly in the vascular endothelial cells and the renal tubular epithelium, it has also been detected in the lungs and the GI tract. Therefore, SARS-CoV-2 can then spread to other digestive organs, such as the liver, by using the same ACE-II enzyme [18,19]. Although no clinical studies have examined the release of the cytokine in the GI tract of COVID-19 patients, studies have shown that upregulation of G protein-coupled receptors occurs in COVID-19 patients, which leads to elevation of inflammatory cytokines [20]. Some of these cytokines have been previously shown to be involved in GI health and pathology such as interleukin-6 (IL-6). The elevation of the cytokines level was interpreted as a protective mechanism to COVID-19 and damage to the GI system [21].

Despite the current proposed theories explaining the pathogenesis of AP manifestation in COVID-19 positive patients, no definitive studies have been conducted to establish the association of the two pathologies. The cohort studies detailed in this paper were too small to be generalized to a larger population.

Previous studies have demonstrated that AP is sometimes medication induced rather than idiopathic. Over 525 different drugs are listed in the WHO database as suspected of causing acute pancreatitis as a side effect. Specifically, drugs used in treating COVID-19 can, directly or indirectly, cause pancreatic injury. Antibiotics, NSAIDS, propofol, and steroids can play a role in the pathogenesis of AP [22,23]. Hypertriglyceridemia is another established etiological factor not frequently considered, and hypertriglyceridemia-associated drug-induced AP was observed in association with the above drugs being used in COVID-19 positive cases [24]. This factor was explained in Kumar V et al. [14], where all mechanically ventilated patients received propofol for sedation at a median dose of 75 μg/kg/min (30–83 μg/kg/min) and for a median of 86 h. Triglyceride levels >500 mg/dL during hospitalization were observed in six patients while three patients had levels >1000 mg/dL. Although triglyceride peak preceded the diagnosis of AP among these patients, the triglyceride levels trended down rapidly in these patients upon discontinuation of propofol and/or tube feeds. The triglyceride levels were only modest at the time of diagnosis of AP.

Most of these studies were performed when SARS-CoV-2 was a new viral disease, and its management was not known to physicians all over the world. Therefore, pancreatic enzymes were not measured in all COVID-19 positive patients unless those patients presented with symptoms suggestive of GI compromise, which would prompt collection of amylase and lipase levels. Finally, 10 studies were reviewed above with the true study result of rare to infrequent AP occurrence in COVID-19 positive patients. These studies are not enough to establish AP and COVID-19 association and outcome.

Interestingly, recent literature reviews in Correia de Sá T et al. [25] and Jabłońska B et al. [26]—where they used a different search approach and included case reports/series—concluded that there is still insufficient evidence demonstrating that COVID-19 can cause AP or negatively impact disease prognosis. Ultimately, the most important clinical research questions are how to predict the course of the disease and the overall outcome when COVID-19 and AP coexist. 

To date, there is no specific treatment suggested in the literature, and we agree with Jabłońska B et al. [26] that the approach is mainly conservative with resuscitating body volume status using intravenous fluid, bowel rest, analgesia, antibiotics, and avoiding medications that can contribute to further pancreatic injury. There have been no human studies evaluating tocilizumab, a monoclonal antibody that competitively inhibits the binding of IL-6 to its receptor, in the treatment of acute pancreatitis [27].

## 5. Conclusions

In conclusion, current data highlight that SARS-CoV-2 infection has been manifesting in various extrapulmonary organs such as GI, heart, and kidneys. The association between acute pancreatitis and COVID-19 has not been clearly established. The ACE-II or cytokines mediated pathogenesis processes are examples of suggested theories explaining a possible causality of AP associated COVID-19 cases. The association between COVID-19 and AP is not based on strong evidence or research. Thus, solid multicenter epidemiological studies are needed to better address the correlation of pancreatic enzymes and AP associated COVID-19 presentation after eliminating other co-founders, including medications used in COVID-19 treatment, to clarify its spectrum of severity and eventually foresee its outcome. 

## Figures and Tables

**Table 1:** Summary of studies discussing acute pancreatitis association with COVID-19.

Study	Subjects	Pancreas Assessment	Total No.	Total No. of COVID-19 Pancreatitis
Wuhan (5)	COVID-19 pneumonia	Elevated lipase (>70 U/L) or amylase (>90 U/L)	52	9
Greenberg (6)	All admissions	Lipase elevation (>3x ULN), scan evidence, and abdominal pain	48,012	189 (32 with COVID-19)
US centers (9)	COVID-19 hospitalizations	Lipase elevation (>60 U/L)	71	9
Two Wuhan Hospitals (10)	COVID-19 pneumonia	Elevated lipase (>78 U/L) and amylase (>135 U/L)	64	23
Rush University-Chicago (11)	COVID-19 positive admissions	Elevated lipase (>3x ULN or >156 U/L)	83	58
Mass General Brigham Healthcare (12)	COVID-19 positive admissions	Elevated lipase (> 3x ULN) and imaging	985	17
Tertiary care hospital-New Delhi (13)	COVID-19 positive admissions	Elevated lipase (>3x ULN, normal range 0–70 U/L) and amylase (>3x ULN, normal range 0–90 U/L)	42	1
A prospective observational-single center study (14)	COVID-19 positive dmissions	Elevated lipase (more than 3 folds of upper limit 300 IU/L) and amylase (more than 3 folds of upper limit 53 IU/L)	110	None
Liverpool (15)	Acute pancreatitis	Clinical review of charts and imaging	35	5
MHealth Fairview- Minnesota (16)	All admissions	Established diagnosis of acute pancreatitis	339	14
Dutch university hospitals-Amsterdam (17)	COVID-19 positive admissions	Established diagnosis of acute pancreatitis	433	5

x: times.
